# The experiences of mothers with preterm infants within the first-year post discharge from NICU: social support, attachment and level of depressive symptoms

**DOI:** 10.1186/s12884-020-02956-2

**Published:** 2020-04-29

**Authors:** Patricia Leahy-Warren, Chelsea Coleman, Róisín Bradley, Helen Mulcahy

**Affiliations:** School of Nursing and Midwifery, University College Cork, Brookfield Health Sciences Complex, College Road, Cork, T12 AK54 Ireland

**Keywords:** NICU, Preterm, Mothers, Postnatal depression, Mother-infant attachment, Social support

## Abstract

**Background:**

The estimated global premature birth rate for 2014 was 10.6%, equating to an estimate of 14.84 million live premature births. The experience of premature birth does not impact solely on the infant and mother as individuals but occurs in the context of a critical point in time when they are developing a relationship with one another. The aim of this study was to investigate the relationships between social support, mother to infant attachment, and depressive symptoms of mothers with preterm infants within the first 12 months’ post discharge from the Neonatal Intensive Care Unit (NICU).

**Methods:**

A correlational cross-sectional study design was used. Data were collected using a four-part online survey which included the Perinatal Social Support Questionnaire (PICSS), Maternal Postnatal Attachment Scale (MPAS) and the Edinburgh Postnatal Depression Scale (EPDS) with mothers of preterm infants (*n* = 140).

**Results:**

The prevalence of postnatal depression was 37.9% (95% CI: 29.8 to 46.4%). In univariable analyses, history of depression (*p* = 0.005), aged 35–39 years (*p* = 0.006), no formal social support (*p* = 0.040), less informal social supports (*p* = 0.018), lower overall maternal attachment (*p* < 0.001) and lower overall functional social support (*p* < 0.001) were significantly associated with a higher level of depressive symptoms. Lower scores on two of the maternal attachment subscales (quality of attachment and absence of hostility) and all four of the functional social support subscales were significantly associated with a higher level of depressive symptoms (*p* < 0.001 for all). In the multivariable analysis, prior history of depression (*p* = 0.028), lower score of maternal attachment (*p* < 0.001) and lower emotional functional social support (*p* = 0.030) were significantly associated with a higher level of depressive symptoms.

**Conclusion:**

Women who experience a premature birth, have a prior history of depression, poor infant attachment and poor emotional social support have a higher level of depressive symptoms. Results emphasise the need for professionals to encourage mobilisation of maternal formal and informal social supports. It is important to intervene early to address maternal emotional well-being and enhance the developing mother-preterm infant relationship.

## Background

### Social support

It is widely accepted throughout the literature that social support enhances the transition to new motherhood [1]. Women have been found to explicitly express a need for professional support that addresses their physical and psychological needs when undergoing the transition to motherhood [2]. Similarly, for parents experiencing neonatal care, a survey conducted in the UK found that personal qualities of staff were of primary importance to parents. Parents in this survey reported that ‘the personal touch of considerate and sensitive staff made all the difference’ [3] p. 16. For mothers of premature infants findings from a number of studies demonstrate that formal social support structures can aid the transition to motherhood [4]; promote maternal confidence [4]; help overcome fears [5]; and support the developing parent to infant relationship [5].

Despite these findings, once discharged from hospital, mothers of premature infants are less likely to access formal support structures than mothers of term infant [6]. Therefore, it is thought that informal support structures play a fundamental role within the family unit. From the limited research available, it is thought that informal social support helps the transition to motherhood by improving mood [7], reducing the experience of isolation [8]; increasing confidence [4, 9] and supporting opportunities for bonding [10]. One study found that social support from significant other/s was vital for the emotional wellbeing of mothers with premature infants [11]. Furthermore, findings suggested that positive affirmations and encouragement from family members, led to positive feelings amongst mothers of preterm infants and helped them identify as mothers [11].

### Mother – preterm infant attachment

Infants born prematurely are a vulnerable population at increased risk of adverse medical, cognitive, developmental and behavioural outcomes [12–14]. The estimated global premature birth rate for 2014 was 10.6%, equating to an estimate of 14.84 million live premature births. The complexities of premature birth occur at a period in time where mothers and infants are expected to bond and form an attachment [15, 16]. Winnicott described the maternal psychological state of late pregnancy and the early post birth period under the term primary maternal preoccupation. This refers to a period when mothers are preoccupied with their infants. During this time mothers may be observed gazing, stroking, touching, feeding and holding their infants, all of which are critical to forming a secure attachment [17].

With reference to Bowlby’s theory of attachment [18], preterm infants can be considered a high-risk group for insecure attachment, because of the potential for prolonged separation. Due to increased risk of medical complications, it is often necessary to remove a baby from their mother immediately at birth for observations and interventions [6, 19]. Thus, their first sensory experience of the world can differ significantly from that of a healthy infant born at term, whose physiological and emotional need to be close to their parents can be met through unlimited skin to skin contact for at least an hour following birth. This gently transitions the newborn to the world, with the supporting touch of their parents to comfort, reassure and co-regulate [20]. Infants depend on their caregiver to help them sooth and internalise the belief that emotions can be calmed; this process is referred to as co-regulation. Co-regulation from a caregiver supports the development of secure attachment; a skill that is necessary for coping with emotions across the lifespan. In an attempt to offer care that recognises infants’ early social and emotional development, various supports have been introduced to many Neonatal Intensive Care Units (NICU). These include: kangaroo care; infant massage; family centred care, which can benefit parent-infant bonding and attachment, thus reducing stress for mothers, and promoting improved developmental outcomes for infants [21–24]. Despite such initiatives, the physical health of infants continues to take precedence over psychosocial domains of health, resulting in preterm infants’ outcomes being compromised [6, 10, 25]. These include: increased irritability, difficulties settling into routines, decreased ability to play and poorer developmental outcomes when compared to infants born at term [10, 15].

### Postnatal depression

Not only is the premature infant faced with such turmoil [15], a parallel process occurs for the mother of a premature infant who is also faced with a number of biopsychosocial factors that can interrupt her transition to new motherhood [4, 19, 26]. Mothers may have to endure the trauma and physical consequences of experiencing an emergency birth, as well as potentially being psychologically unprepared for the abrupt arrival of her newborn [19]. This can evoke a variety of emotional responses including sadness, failure, fear and uncertainty. Unsurprisingly, women who experience premature birth are at increased risk of ill health [6], including an increased risk of depressive symptoms [27]. In addition to this, mothers with compromised psychological health may feel less invested in their relationship with their newborn infant, thus affecting opportunities for bonding and therefore forming a secure attachment [27].

Prevalence of postnatal depression (PND) across all births vary from 10 to 15%, depending on the screening instrument, timing and cut-off scores used [28]. Mothers of infants born prematurely have almost double the rates of PND (28–40%), in the early postpartum period [29]. There is a paucity of research, examining how these rates continue to be affected within the first-year post discharge from hospital, with most of the research concentrating on the early postnatal period. For mothers of healthy term infants it has been well established that structural support both formal and informal, reduces the risk of PND [1, 30–32]. However, a paucity of research exists examining how formal and informal social support relate to the level of depressive symptoms amongst mothers of premature infants.

## Method

### Aims

The aim of this study was to investigate the relationships between social support, mother to infant attachment, and depressive symptoms, of mothers with preterm infants within the first 12 months’ post discharge from NICU. To meet this aim this study set the following three objectives:

1. To understand mothers’ subjective experiences of attachment towards their infants and the availability of social support in the first-year post discharge from NICU.
2. To establish level of depressive symptoms amongst mothers of preterm infants within the first year postpartum.
3. To the examine the relationships between social support, mother to infant attachment and depressive symptoms.

### Design

A correlational cross-sectional design was used.

### Subjects

A convenience sample of mothers of preterm infants discharged from NICU, within one year formed the sample. Although a lack of randomization is a weakness to convenience sampling, this was the most appropriate method of sampling to reach the specific population subgroup and achieve the study aims and objectives. In collaboration with a statistician and after undertaking a power analysis it was deemed appropriate that a minimum of 84 participants were needed for the study. A total of 140 participants who met the inclusion criteria and completed all questions on the survey were suitable for analysis. This cross-sectional study was conducted in December 2016. The inclusion criteria were as follows: mothers who had premature infants admitted to NICU within past 12 months; could read and understand English; at least 18 years old; had access to internet with parenting and preterm infant websites. The exclusion criteria were as follows: mothers of preterm infants that have died; mothers of preterm infants that were ‘in care’ with social work involvement; mothers of preterm infants who did not spend any time in the NICU.

### Procedures

Prior to data collection, ethical approval was sought and granted by the local Clinical Research Ethics Committee, REF: ECM 4(b) 06/09/16. Data were collected by means of an online survey using Survey Monkey (Survey-Monkey.com). This software provided a secure channel for collecting data online and offered a facility for documenting consent separately, therefore anonymity was upheld at all times. In order to gain consent, mothers of preterm infants were asked to indicate their willingness to partake in the study by selecting yes/no of Question 1 in Section 1 of the online survey. Participants were provided with information on the aims, objectives of the study and inclusion and exclusion criteria for participants. Mothers were also made aware of their right to withdraw from the study at any time point. This consent procedure was approved by the relevant ethics committee.

Prior to obtaining a full data collection, a pilot study was undertaken with online respondents (*n* = 13). This allowed for assessment of questions for the clarity and the length of time for completion. No changes were recommended by respondents. To facilitate a high response rate, the URL link was posted on several relevant Irish websites and Facebook pages such as ‘Premature Infant Ireland’ and the ‘Irish Multiple Birth Association’. Once the link to the study was opened, participants were invited to part take in the study through an introductory section and were made aware of their right to withdraw by closing Survey Monkey©.

### Measurements

All data were collected using a four-part online survey which included a demographic questionnaire; The Perinatal Infant Care Social Support (PICSS) questionnaire; The Maternal Postnatal Attachment Scale (MPAS); and the Edinburgh Postnatal Depression Scale (EPDS).

The PICSS questionnaire was used to measure mothers’ self-reported structural (formal and informal social networks) and functional (informational, instrumental, emotional and appraisal) dimensions of social support in the context of infant care [1, 30]. Self-reported functional social support was measured using 22 items on a 4-point Likert scale. Scores range from 22 to 88, with higher scores indicating greater availability of social support. The functional support scale has four subscales: “informational support” (7 items, possible range of scores: 7 to 28); “instrumental support” (7 items, possible range of scores: 7 to 28), “emotional support” (4 items, possible range of scores: 4 to 16), and “appraisal support” (4 items, possible range of scores: 4 to 16). Structural social support is a list of individuals who can provide support to mothers. The list includes both informal (i.e. husband/partner, mother) and formal (i.e Midwife, Nurse, GP,) sources of support. Participants are then asked to select the type of support (informational, instrumental, emotional or appraisal) from each source. An informal structural support score was obtained by summing the number of informal sources that a participant had available to them. The availability of formal structural social support was reported if the woman had at least one source of formal support [30]. The development and psychometric testing of the Perinatal Infant Care Social Support (PICSS) instrument has since been further developed and found to be a coherent and valid measure of social support for new mothers in the perinatal period, in the context of infant care practices [33].

MPAS [34] consists of 19 statements on a Likert scale to measure participants’ self-assessed attachment to their infant in the first year post discharge from NICU. As stated by Condon (1998) this scale is exclusively concerned with the mothers’ self-assessment of her attachment to her infant and unlike previously conducted observational studies [34]. Scores on the scale can range from 19 to 95, with higher scores indicating greater self-report mother to infant attachment. MPAS has three subscales: “quality of attachment” (9 items, possible range of scores: 9–45); “absence of hostility” (5 items, possible range of scores: 5 to 25) and “pleasure in interaction” (5 items, possible range of scores 5 to 25). Total MPAS scores were calculated by summing the subscale scores.

Level of depressive symptoms was measured using the EPDS [35], which consists of 10 items on a 4-point Likert Scale. Scores can range from 0 to 30, with a cut-off of 13 or more used to identify women with an increased level of depressive symptoms.

### Statistical analysis

All statistical analysis was performed using IBM SPSS Statistics (version 25.0, IBM Corp, Armonk, NY, StataCorp U.S.A.). Categorical variables were described using frequencies and percentages. Continuous variables were described using mean and standard deviation (SD).

Univariable and multivariable linear regression models were used to investigate factors associated with level of depressive symptoms. Independent variables with a *p*-value< 0.25 in the univariable analysis were eligible for inclusion in the multivariable analysis. For the multivariable analysis, if the subscales for MPAS and PCISS explained more of the model variance than the overall scales, the subscales were included in the model rather than the overall scales. Only participants with information on all of the variables of interest are included in the analysis. Reliability of the MPAS, PICSS functional social support scales and subscales and the EPDS scale were assessed using Cronbach’s alpha. The items within the structural social support scale and subscales are not necessarily correlated and hence reliability statistics are not applicable. All tests were two-sided and a *p*-value< 0.05 was considered to be statistically significant.

## Results

### Socio-demographic and infant characteristics

A total of 140 participants provided information on all variables of interest. Participants’ socio-demographic and infant characteristics are shown in Table 1. The majority of participants were aged 30–39 years (71.4%), educated to third level (76.4%) and not-first-time mothers (55.0%). No questions relating to the ethnicity/nationality of participants were asked. However, as the survey link was shared on Irish websites and Facebook pages, it is likely that most participants were living in Ireland. Over three-quarters (76.4%) of participants gave birth to a singleton infant and the majority (59.3%) were moderate to late pre-terms. The median (IQR) age of the children at the time of the survey was 7.0 (4.6 to 11.5) months. This is based on 138 children for whom the data on age was available. One-fifth of participants (20.0%) reported having a prior history of depression. The majority of participants fed their infants breast milk in NICU (87.1%), with almost half exclusively feeding breast milk (45%). Only 12.9% of participants reported that they exclusively fed their infants infant formula milk in NICU.

**Table 1:**
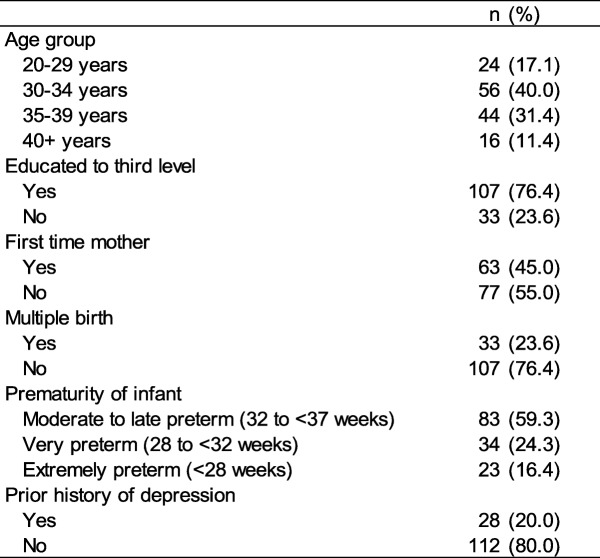
Participants’ (*n* = 140) sociodemographic and infant characteristics

### Mother-preterm infant attachment, social support and postnatal depression

Descriptive statistics for social support, mother to preterm infant attachment and level of depressive symptoms are shown in Table 2. The number of informal sources reported by participants ranged from 1 to 10 with a median (IQR) of 6 (4 to 8). The persons most frequently identified by participants were husbands/partners (92.1%), friends (83.6%) and own mothers (80.7%). The majority of participants (88.6%) reported having at least one source of formal social support and the public health nurse was reported most frequently (80.0%), followed by GP (65.7%), midwife (62.1%) and practice nurse (36.4%).

**Table 2:**
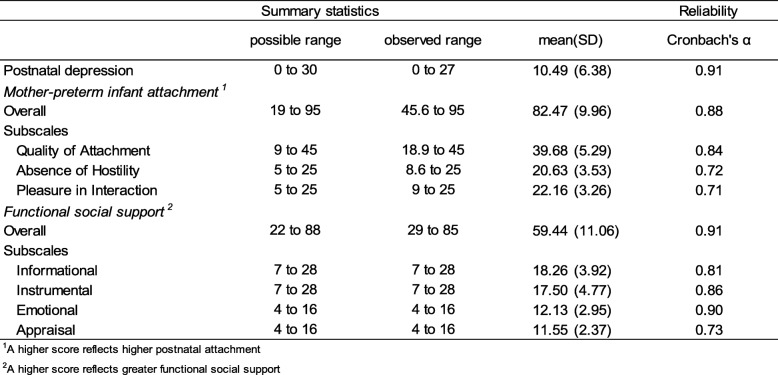
Descriptive Statistics and Reliability of Instruments (*n* = 140)

^1^A higher score reflects higher postnatal attachment. ^2^A higher score reflects greater functional social support

The mean total and subscales scores on the MPAS indicated high mother to infant attachment. The mean total and subscales scores on the functional PICCS indicated that participants reported high levels of all four functional social supports.

EPDS scores ranged from 0 to 27 with a mean (SD) of 10.49(6.38). Of the 140 participants, 53 scored 13 or higher, giving a risk of PND prevalence of 37.9% (95% CI: 29.8 to 46.4%).

### Factors associated with depressive symptoms

The results of the univariable analyses are presented in Table 3. From demographic and infant characteristics, maternal age group (*p* = 0.006) and prior history of depression (*p* = 0.005) were significantly associated with level of depressive symptoms. Women aged 35–39 years had a significantly lower level of depressive symptoms compared to women aged 20–29 years (*p* = 0.007) and those aged 30–34 years (*p* = 0.037) and having a prior history of depression was associated with a higher level of depressive symptoms post preterm birth.

**Table 3:**
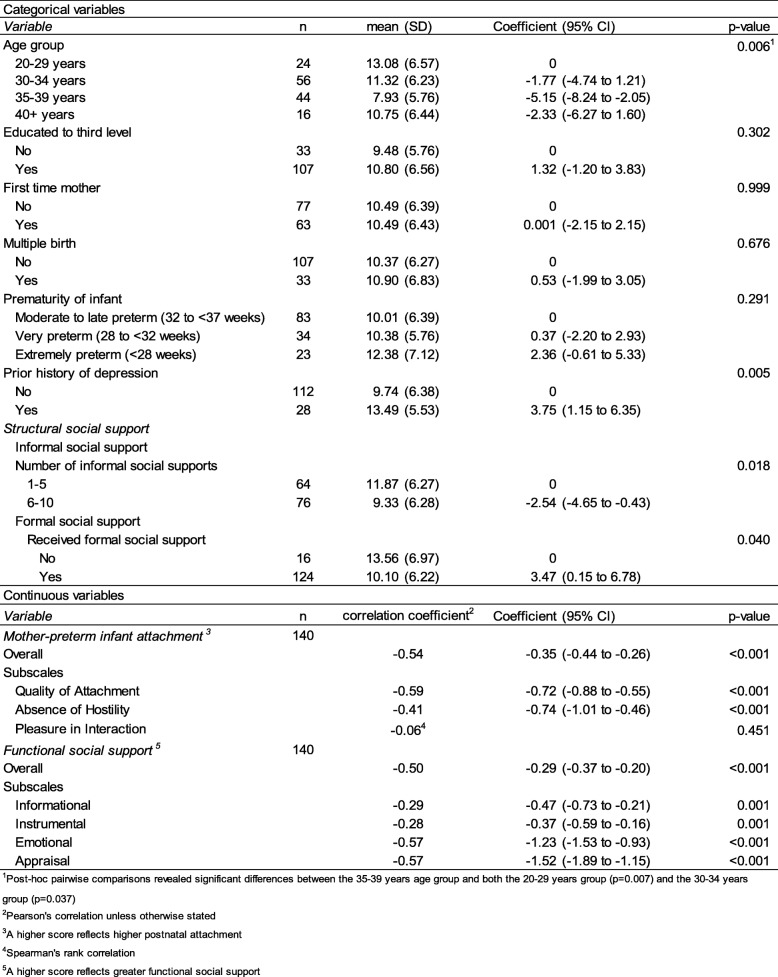
Univariable analyses investigating factors associated with EPDS, *n* = 140

^1^Post-hoc pairwise comparisons revealed significant differences between the 35–39 years age group and both the 20–29 years group (*p* = 0.007) and the 30–34 years group (*p* = 0.037). ^2^Pearson’s correlation unless otherwise stated. ^3^A higher score reflects higher postnatal attachment. ^4^Spearman’s rank correlation. ^5^A higher score reflects greater functional social support

There was a statistically significant negative relationship between self-assessed attachment and level of depressive symptoms (*p* < 0.001), indicating that having alower level of attachment was associated with high depressive symptoms. Statistically significant relationships were found between two of the attachment subscales: quality of attachment (*p* < 0.001) and absence of hostility (*p* < 0.001) and level of depressive symptoms. No significant relationship was found between pleasure of interaction subscale and level of depressive symptoms (*p* = 0.451).

There was a statistically significant negative relationship between total functional social support and level of depressive symptoms (*p* < 0.001), indicating that having greater functional support was associated with having lower depressive symptoms. Statistically significant relationships were found between all functional social support subscales and level of depressive symptoms: informational, instrumental. Emotional and appraisal support (*p* < 0.001 for all). Number of sources of informal social supports (*p* = 0.018) and receiving formal social support (*p* = 0.040) were significantly associated with level of depressive symptoms. Having 6–10 sources of informal social support (versus 1–5) and receiving formal social support were associated with a lower level of depressive symptoms.

In the multivariable analysis (see Table 4), prior history of depression (*p* = 0.028), quality of self-reported attachment (*p* < 0.001) and emotional support (*p* = 0.030) remained significantly associated with level of depressive symptoms. Women with a prior history of depression, lower self-assessed attachment and lower emotional support had a higher level of depressive symptoms.

**Table 4:**
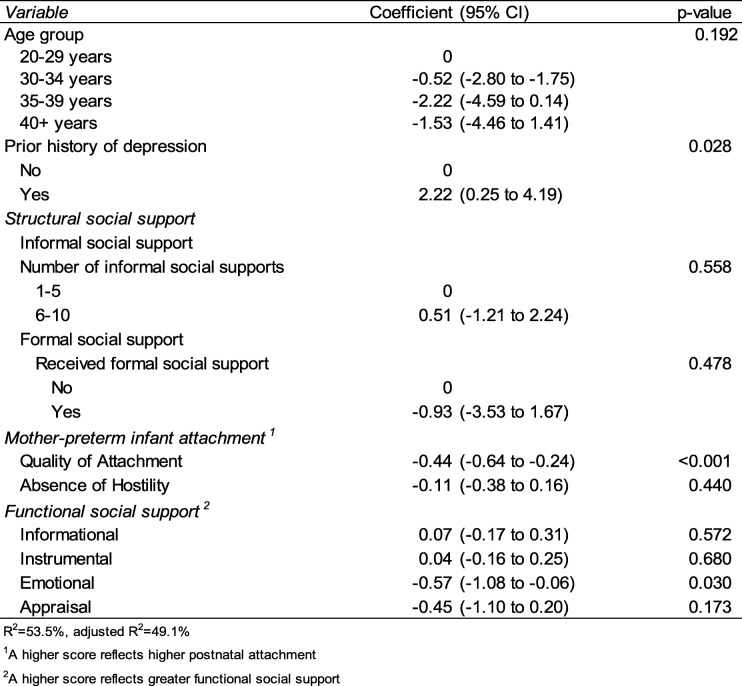
Multivariable analysis investigating factors associated with EPDS, *n* = 140

R2 = 53.5%, adjusted R2 = 49.1%. ^1^A higher score reflects higher postnatal attachment. ^2^A higher score reflects greater functional social support

## Discussion

Results from this study reveal that mothers who gave birth to premature infants were at a 38% increased risk of PND within the first-year post discharge from NICU. Maternal age (20–34-year olds) and a reported history of depression were statistically associated with a higher level of depressive symptoms. These findings are clinically important as little is known about preterm mothers’ depressive symptoms within the first-year post discharge from NICU. Although, these results are in line with findings from other studies [6, 29], the time period is unique. Previous studies examining risk of PND amongst mothers of healthy term infants, have suggested that the prevalence of risk of PND decreases as the postnatal period progresses [1]. However, when this survey was completed the age of the infants were between 4.6 months and 11.6 months. This suggests that mothers of preterm infants have a higher level of depressive symptoms for an extended period postpartum. Prior to hospital discharge universal screening for depressive symptoms amongst mothers of preterm infants needs to be considered as part of routine care. Further screening and follow up care should be determined by the results of this initial screen. As recommended by Mounts, research is needed to identify the most appropriate screening programme for women whose infants have been admitted to the NICU, and to establish the optimal time for screening amongst a population where little is known about their course of depression [36].

A systematic review exploring the risk of PND amongst mothers of preterm and low birth weight (LBW) infants concluded that, mothers of very premature and very LBW infants have higher rates of depressive symptoms, throughout the first year postpartum, with very limited reduction in symptoms [19]. Interestingly, most of the infants (60%) in this study had a gestation of 32 weeks or more at time of birth, suggesting that an increased risk of PND within the first-year postpartum is not exclusive to those with very premature or very LBW infants. A potential rationale for this could be that preterm birth can interrupt the woman’s psychological processes of building representations of the child and of oneself as a mother, thus hindering her transition to motherhood and impacting on her emotional wellbeing [19]. Further qualitative research is needed, to explore possible reasons for the prolonged increased risk of PND amongst mothers of preterm infants, within the first-year post discharge from NICU.

Although this study found that mothers’ self-assessed attachment to their infant is not necessarily compromised by NICU admission. There is growing awareness of the importance of enhancing the parent-infant relationship within NICU [37]. Findings from this study emphasise the need for increased awareness for healthcare staff and parents surrounding the impact of prematurity on attachment and level of depressive symptoms.

Initiatives supporting such awareness need to focus on community-based healthcare providers, as they were found to be the professionals most available to women for functional support following hospital discharge. Increased awareness surrounding the key concepts of infant mental health have the potential to support practitioners to provide shared attention to the infant, the parent and the developing relationship; whilst also providing families with the necessary skills to support bonding and attachment [38]. Alderdice & Redshaw suggests the use of early interventions, such as the use of the Newborn Observational Tool, to support psychosocial assessment and encourage professionals to work directly with families to support early parent child interactions and engagement [37].

Mothers reported having a high level of functional social support, which was associated with lower level of depressive symptoms. Having greater informal structural social support (e.g. partner) (6–10 v 1–5) and having at least one source of formal structural support (e.g. PHN) was also associated with lower level of depressive symptoms. Increasingly, research is emphasising the need for health services to consider and include mothers’ key social supports in the care provided [39]. The benefits of family centred care and developmental care packages within NICUs, that ensure the whole family feel welcomed and valued, are becoming increasingly recognised [40]. This study further highlights the importance of including the mother’s wider support structures in the care provided in hospital, as it is evident that mothers depend on these support structures within the first-year post discharge from NICU. Furthermore, results highlight a need for health care professionals to include parents’ significant others within the provision of primary care and community-based support services. A study conducted in the UK, highlighted that mothers of preterm infants were less likely to access support once discharged from hospital [6]. This further affirms the need for professionals to involve family members in the community-based care, as it has the potential to enable the availability of indirect support to the mother and infant, who may not directly access formal sources of support.

This study has some limitations such as the sample selection and the design. A convenience sample was necessary in order to access such a hard to reach sample. A random sample may have provided a group with differing support needs, self-assessed attachment level and level of depressive symptoms. However, a complete sampling frame would not have been possible for parents of infants of this desirable age. Online recruitment has inherent limitations; however, it does permit access to a sample previously inaccessible. Finally, all those participants that returned completed questionnaires and met the inclusion criteria were included, therefore, these participants are representative of mothers of preterm who respond to online surveys and not the general population and thus a limitation of the study. Further research is needed whereby innovative research methods are used to include women from societal groups that are less likely to be able to complete online English surveys due to socioeconomic; language; mental health and educational barriers.

## Conclusion

To conclude, significant relationships were found between age and previous history of depression and depressive symptoms; self-assessed attachment and depressive symptoms; total functional social support and depressive symptoms; having more sources of informal social support and depressive symptoms and having formal social support and depressive symptoms. Furthermore, women with a prior history of depression, low self-assessed attachment and low emotional support had a higher level of depressive symptoms. Recognition and understanding of how these variables interact at this critical period for infant development is fundamental to ensuring emotional, social and physical health and wellbeing of both mother and infant.

Women who experience a premature birth, have a prior history of depression, poor infant attachment and poor emotional social support have a higher level of depressive symptoms. Results emphasise the need for professionals to encourage mobilisation of maternal formal and informal social supports. It is important to intervene early to address maternal emotional well-being and enhance the developing mother-preterm infant relationship.

## Data Availability

Public access was not part of the ethical approval for this research study, thus data and materials are not available.

## Abbreviations

EPDS: Edinburgh Postnatal Depression Scale
MPAS: Maternal Postnatal Attachment Scale
NICU: Neonatal Intensive Care Unit
PICCS: Perinatal Infant Care Social Support
PND: Postnatal Depression
